# Patient experiences of receiving a diagnosis of Parkinson’s disease

**DOI:** 10.1007/s00415-018-8817-8

**Published:** 2018-03-15

**Authors:** A. Schrag, S. Modi, S. Hotham, R. Merritt, K. Khan, L. Graham, A on behalf of the European Parkinson’s Disease Association

**Affiliations:** 1 Department of Clinical Neurosciences, UCL Institute of Neurology, Royal Free Campus, University College London, London, NW3 2PF UK; 2 Centre for Health Services Studies, University of Kent, Canterbury, UK; 3 European Parkinson’s Disease Association, Brussels, Belgium

**Keywords:** Parkinson’s disease, Patient-reported outcome, Diagnosis, Communication, Experience

## Abstract

**Objective:**

To report patients’ own experiences of receiving a diagnosis of Parkinson’s disease (PD) and to identify factors influencing this experience.

**Methods:**

A survey by the European Parkinson’s Disease Association in 11 European countries.

**Results:**

1775 patients with an average age of 69.7 years participated of whom 54% were male. Those living in rural areas reported having waited longer to seek medical help (*p* < 0.05). A possible diagnosis of PD was made at the first appointment in a third of respondents. When the diagnosis was made, only 50% reported that the diagnosis was communicated sensitively. 38% of patients reported having been given enough time to ask questions and discuss concerns, but 29% did not. 98% of participants reported having been given information about PD at the time of diagnosis but 36% did not find the information given helpful. Patient satisfaction with the diagnostic consultation was positively associated with more sensitive delivery of diagnosis, the helpfulness and quantity of the information provided and time to ask questions (all *p* < 0.001). Where diagnosis was given by a specialist, participants reported greater perceived satisfaction with the diagnostic consultation, greater sensitivity of communicating the diagnosis, time to ask questions, provision and helpfulness of information, and earlier medication prescription (all *p* < 0.0001).

**Conclusions:**

There is a need to improve how the diagnosis of PD is communicated to patients, the opportunity to ask questions soon after diagnosis, and the amount, timing and quality of life information provided, as this is associated with greater satisfaction with the diagnostic process.

**Electronic supplementary material:**

The online version of this article (10.1007/s00415-018-8817-8) contains supplementary material, which is available to authorized users.

## Introduction

The experience of receiving a diagnosis of Parkinson’s disease (PD) has been reported to have a significant impact on patients’ quality of life even many years after the initial diagnosis [1]. Several aspects of the diagnostic process, including time to diagnosis, the referral process, how it is reached, the way it is communicated, the information provided and explained, the follow-up actions planned and the treatments started are all likely to be factors that can influence the impact of the diagnosis [2]. However, how the diagnostic process is experienced from the patient’s point of view and which factors influence their experience of the diagnostic process have received little attention. To improve the experience of receiving a diagnosis of PD and to mitigate its long-term impact, it is important to understand how patients experience this process and what aspects are of relevance from their point of view. This information will allow health care professionals involved in the diagnostic process improve the experience and impact of this diagnosis for patients.

We here report the results of a large survey in people with PD from 11 European countries on their subjective experiences of receiving a diagnosis of PD.

## Methods

Between 1st November 2014 and 12th January 2015, the European Parkinson’s Disease Association (EPDA) conducted a survey in patients with PD through its national patient organisations from 11 countries (Germany, France, Holland, Sweden, UK, Ireland, Slovenia, Spain, Italy, Hungary, and Denmark). Participants completed a self-reported online survey (except in Slovenia where, due to low internet access, hard copies of the survey were distributed via the national Parkinson’s Association). This included questions on demographics, disease duration and initial symptoms, on experiences of initial diagnosis and what healthcare professional made the diagnosis, how sensitively the diagnosis was given, opportunity to ask questions, information provided in the consultation, medication prescribed and on satisfaction with the diagnostic consultation (see Supplementary Material).

## Data analysis

Descriptive results are presented as total numbers and percentages and mean with standard deviation (SD) or median (range), if not normally distributed. Correlations were examined using Spearman rank correlations and frequencies compared using Chi-square tests. Groups were compared using Mann–Whitney *U* test and Kruskal–Wallis *H* tests. Significance level was set at 5%. All analyses were conducted using SPSS (versions 21 and 24).

## Results

1775 patients completed the survey. Participant characteristics are given in Table 1. Average age was 69.7 (SD 56.3) years with a median disease duration of 7 (< 1 to 42) years and 54% were male.

**Table 1:** Characteristics of participants in the EPDA survey (*N* = 1775)

	Mean (SD)	Median (range)	Country	Number [*n* (%)]
Male [*n* (%)]	958 (54)			
Age of onset	58.5 (10.0)	59.0 (65.0; 25–90)	UK	110 (6.3)
Years since diagnosis	8.2 (6.1)	7.0 (41.0; 1–42)	Holland	175 (10.1)
Years since symptom onset (%)			Denmark	146 (8.4)
< 1 year	9.4		France	47 (2.7)
1–2 years	8.8		Hungary	66 (3.8)
2–3 years	11.9		Germany	84 (4.8)
3–5 years	17.0		Spain	64 (3.7)
5–10 years	29.7		Slovenia	90 (5.2)
> 10 years	23.3		Italy	151 (8.7)
Employed [*n* (%)]	333 (18.8%)		Sweden	806 (46.3)
			Environment [*n* (%)]	
			Rural	343 (19.4)
			Town	655 (37.0)
			City	774 (43.7)

### Lead-up to diagnosis

29% of participants reported having waited for 12 months or more after first noticing symptoms before seeking medical help, 21% 6–12 months, 17% 3–6 months, 24% less than 3 months and 9% could not remember how long they had waited. Those living in rural areas reported having waited longer to seek medical help than those living in a city or town (*p* = 0.007) with no differences in gender, age and countries, except Slovenia where patients waited longer (*p* = 0.003 compared to the UK).

### Experience of diagnostic consultation

**Fig. 1:**
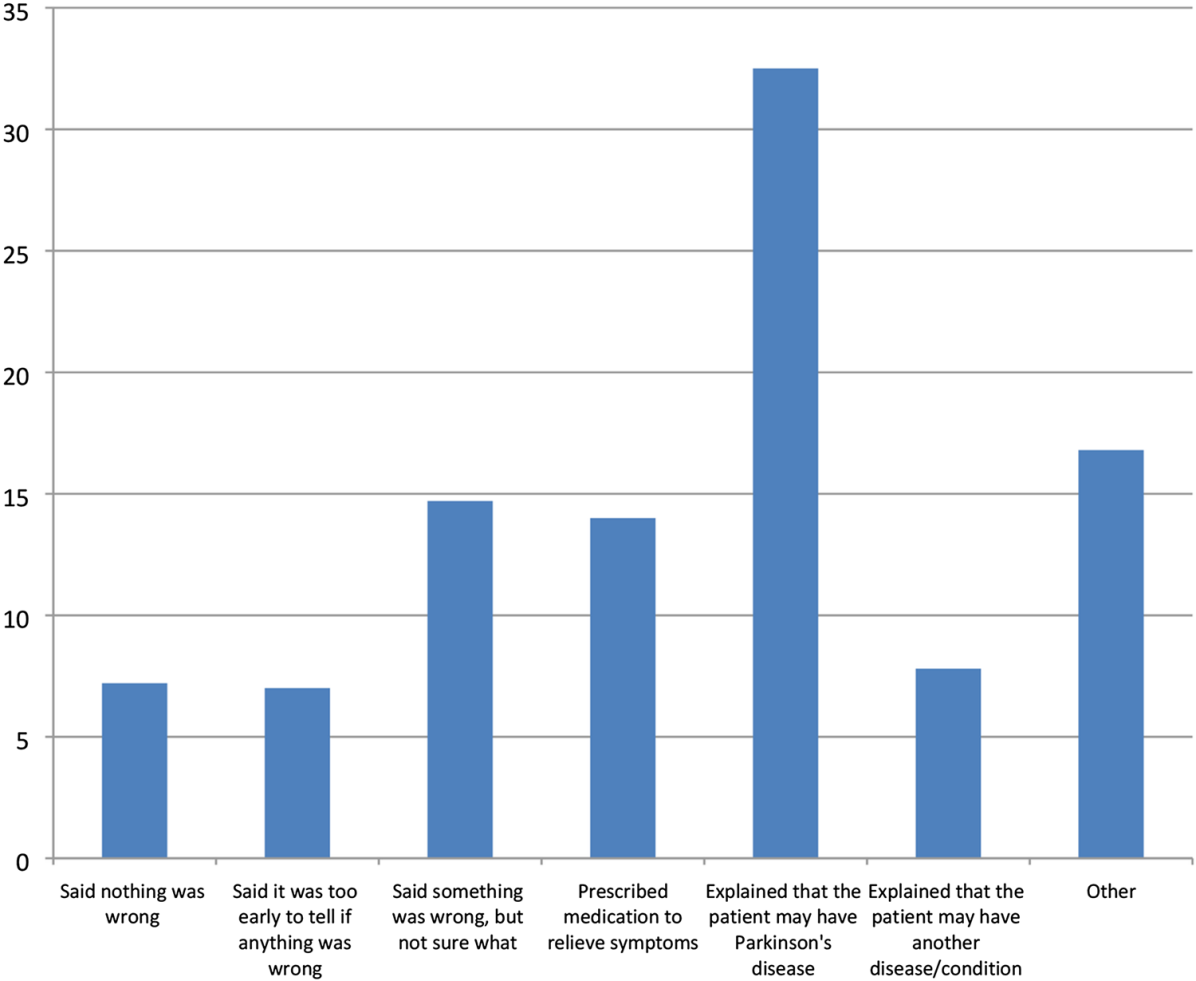
Explanation at first consultation (% of respondents)

**Fig. 2:**
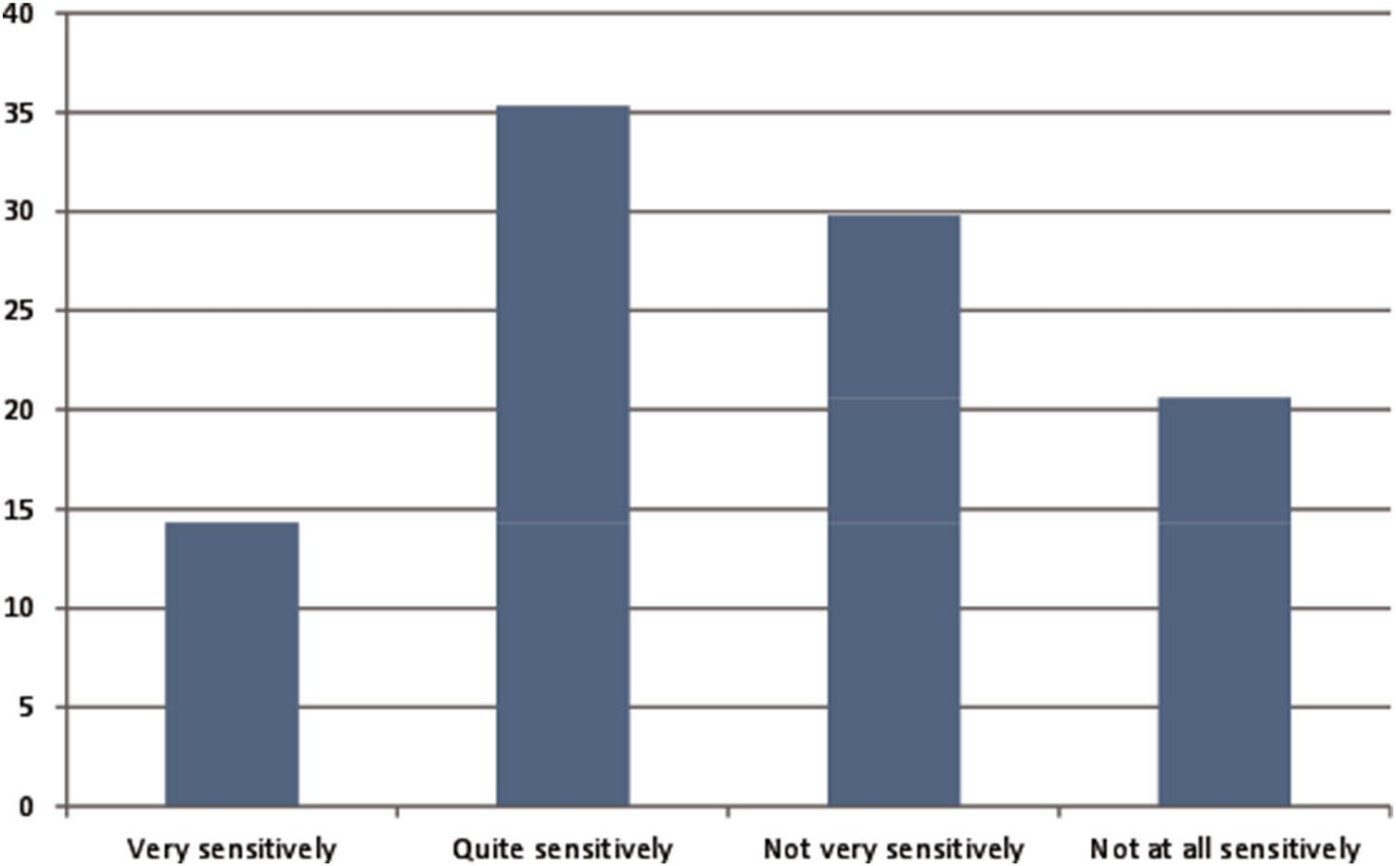
Sensitivity of communication of diagnosis (% of respondents)*

A possible diagnosis of PD was made at the first appointment in a third of respondents of the overall sample with the majority requiring additional appointments (Fig. 1). Eight percent were told they had a different diagnosis and 7% that nothing was wrong during their first appointment with a healthcare professional. Similar responses were reported in those diagnosed aged 50 years or younger and those older than 50 years at diagnosis, and in those who were diagnosed by a specialist or general neurologist (Supplementary Material Fig. 1). The highest numbers reporting being given a possible diagnosis of PD at the first appointment were in Hungary (48%), Holland (42%), Sweden (40%) and Denmark (38%; Fig. 2 Supplementary Material).

The diagnosis of PD was given most commonly by a neurologist, with (52%) and without specialist interest in PD (34%, see Supplementary Material Fig. 3 for country by country). 57% of the overall sample reported having had a brain scan at the time of diagnosis. Higher rates of brain scans at the time of diagnosis were reported in Germany (79%) and Denmark (73%) and lower rates in UK (43%) and France (43%).

**Fig. 3:**
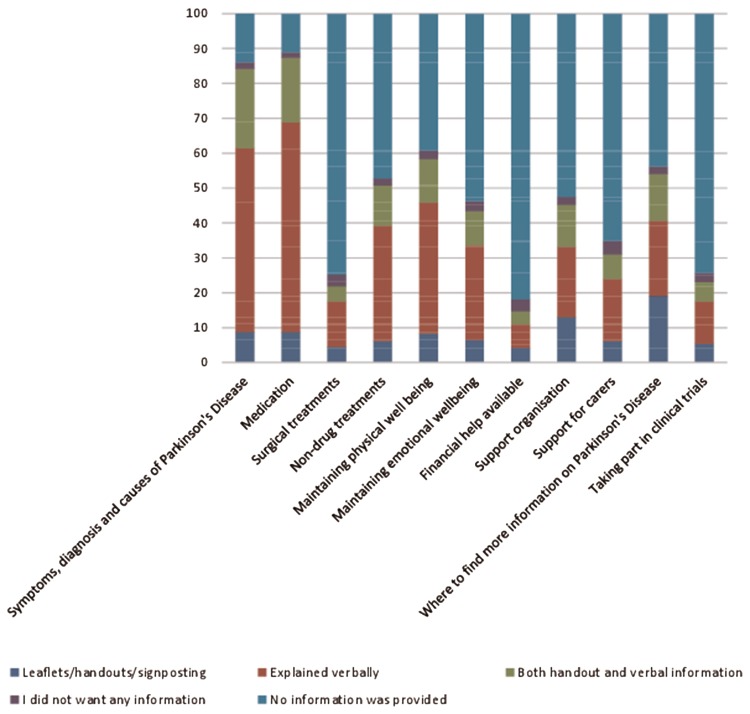
Information received at diagnosis (% of respondents)

Fifty percent of participants reported that they were told they had PD quite or very sensitively, but 50% felt they were told not very or not at all sensitively (Fig. 2). After being given the diagnosis of PD, 38% of patients reported having been given enough time to ask questions and discuss concerns, 17% reported that they would have liked more time to ask questions, 12% reported not having been given any time for questions, 28% did not want or feel able to ask questions at the time and 4% could not remember. However, this was different between countries (*p* < 0.0001, Supplementary Material Fig. 4). The amount of information provided at diagnosis varied, with 2% of participants reporting not receiving any information at diagnosis (Fig. 3). The type and mode of information provided were predominantly verbal information on the disease, with most information on symptoms, diagnosis, causes of PD and medication (Fig. 3). Nearly half of the respondents reported that they had not received any information on non-drug treatments (e.g. physiotherapy) at diagnosis. The information provided was perceived as helpful by 64% of those who were able to provide this information but 36% did not find the information helpful (see Table 2). Treatment was started mostly immediately after diagnosis (67%).

**Table 2:** Reported helpfulness of information received

	*n*	%
Very helpful	220	15.3
Quite helpful	534	37.1
Not very helpful	285	19.8
Not helpful	135	9.4
Cannot remember	269	18.5

### Satisfaction with diagnostic consultation

49 percent of the overall sample reported they were satisfied with the initial consultation and 29% being neutral, but 22% reported being dissatisfied with the consultation at diagnosis. Respondents in Hungary (66%), Slovenia (65%), and Denmark (63%) reported the highest levels of satisfaction with the initial consultation (see Supplementary Material Table 1). Patient satisfaction was strongly associated with more sensitive delivery of diagnosis (*r *= 0.65, *p* < 0.0001) and the helpfulness of the information provided (*r *= 0.52, *p* < 0.0001), and fairly with the time provided to ask questions (*r *= 0.37, *p* < 0.0001), the quantity of information provided (sum of areas for which information was provided, *r *= 0.29, *p* < 0.0001), but correlated only poorly (*r *< 0.2) with age, disease duration, age of onset, how long patients had waited to seek medical attention and how quickly they received medication after diagnosis. Those who received their diagnosis at the first appointment had slightly greater satisfaction with the diagnostic consultation (*p* = 0.05) but there was no difference between participants with different employment status, habitat, gender or those who had a brain scan or not. Where diagnosis was given by a specialist PD neurologist, there was a greater perceived satisfaction with the diagnostic consultation (*p* < 0.0001), sensitivity in delivery of diagnosis, time to ask questions, provision and helpfulness of information and shorter timeframe in which the medication was prescribed (all *p* < 0.0001) compared to general neurologists, geriatricians, hospital doctors and GP/family doctors.

## Discussion

In this large survey in several European countries, almost half of all patients with PD reported that they were not satisfied with initial diagnostic consultation. As the way the diagnosis is communicated has been reported to be associated with quality of life scores even years later [1], improving this experience is important in the care of patients also in the longer term. Demographic factors or where participants lived did not have a strong influence on how satisfied they were with the diagnostic consultation, although those living in rural areas and patients in Slovenia had waited longer to seek medical attention. A diagnosis of PD was made at the first appointment in a third of patients and in approximately a third there was diagnostic uncertainty following the first appointment (likely to reflect true diagnostic uncertainty or specialist confirmation being awaited), but a substantial proportion was initially told they had a different diagnosis or nothing was wrong. There were some differences between countries in experiences at the time of first appointment that are likely to reflect national guidelines as well as health care structures and availability of resources. For example, greater numbers of individuals reported having had brain scans in Germany and France, the highest proportion of patients reported receiving a diagnosis and medication already at the initial consultation in Hungary, and the highest percentage of participants reported having been given enough time to ask questions at the initial diagnosis in Holland. However, whether the diagnosis was made at the first appointment or whether there had been a diagnostic delay only slightly influenced how satisfied patients were with their diagnostic consultation. In addition, whilst we only collected information on participants’ recollection of having had a brain scan and on how quickly medication was prescribed (which, therefore, may not represent accurate information on clinical practice), neither of these factors influenced satisfaction ratings. This suggests that whether a brain scan is performed and whether medication is prescribed immediately are not key factors for the subjective experience of receiving a diagnosis of PD. Of much greater importance to patient satisfaction with the diagnostic consultation was the time patients reported having had at the diagnostic consultation to ask questions, and how much information was provided to them on various aspects of PD and its management. The most important factors, however, for how satisfied patients were with the diagnostic consultation, were how sensitively the diagnosis of PD was communicated to them and the helpfulness of the information about PD provided. These factors were also associated with communication of the diagnosis by PD specialists, who in turn had higher ratings in satisfaction with the diagnostic consultation.

Information on how satisfied patients were with the diagnostic consultation and the factors with the greatest influence on this provides an opportunity to improve the experience of being diagnosed with PD. Whilst health care professionals in diagnostic consultations necessarily need to focus on making the correct diagnosis, ordering appropriate tests and starting medication, subjective patient experience of receiving a diagnosis of PD is primarily determined by how the diagnosis is communicated, the quantity and helpfulness of information is provided and whether they have the opportunity to ask questions. Training in communicating a diagnosis of PD in a sensitive way, on how to provide information verbally and in written form, and giving time to ask questions in the consultation or shortly afterwards are important factors that can shape this experience. Guidelines for the management of PD, e.g. the NICE [3] or EFNS guidelines [4], already incorporate some of these recommendations such as tailored provision of communication, but do not specifically address how to communicate the diagnosis or provide sufficient time for questions on the diagnosis, e.g. in a follow-up or nurse specialist appointment.

Delivering bad or difficult news is an important aspect of how clinicians have influence on the impact of disease on the individual. Surveys in other disorders showed that patients wanted their doctors to be truthful, caring, and compassionate in communication of difficult news [5]. It has also been shown across medical conditions that bad news communicated badly can cause confusion, long-lasting distress, and resentment; if done well, it can assist in understanding, acceptance, and adjustment [6]. Guidelines emphasise the importance of preparation, assessing the patient’s understanding, giving information, follow-up and discussion of treatment options, and assessing patients’ emotions [6]. Most of these guidelines are focussed on cancer or terminal illness, but an increasing emphasis on delivering bad news is also being placed in neurology [7–9] and chronic, non-fatal diseases. In PD, there is still a need to improve how the diagnosis is communicated to patients, in particular the sensitivity of communication in the context of a patient’s understanding, emotions, and expectations. The opportunity to ask questions soon after diagnosis is valued by patients, and the amount, timing and quality of life information provided need to be adjusted to individual patients but improves satisfaction with the diagnostic process. This information, as well as specialist knowledge, is often more easily available to specialists, and the diagnosis is, therefore, best made and communicated by them, followed by a second appointment with a health care professional to allow questions to be asked about a new diagnosis of PD.

## Limitations

As this was a survey on patients’ experiences of diagnosis, with a varying number of years since diagnosis, patients may not always have remembered all aspects of the diagnostic process accurately, particularly as they may not have been able to take in all information given at the time [10]. However, patient experience and its impact were the focus of this paper rather than the actual service provided to allow assessment of factors that can be modified to improve the experience. In addition, the survey was conducted through the national patient organisations online and participants had a relatively young average age; it is, therefore, likely that younger, more educated patients and those who are more active and involved in their management were also more likely to participate in this survey. This may have introduced a bias to patients with greater education and with higher expectations of information provision and of time given to ask questions. However, the sensitivity of how the diagnosis was communicated is unlikely to have been influenced by this. In addition, we did not explore the influence of family and social factors and availability of financial and other societal measures on the experience of receiving a diagnosis. These factors are very likely to be important modifying factors as will be expectations and personality characteristics which we did not explore in this study.

## Conclusions

Whilst referral times and time to ask questions may be influenced by availability of resources in constrained health care systems, many of the factors identified to influence satisfaction with care are not cost intensive, but could be improved by greater awareness and training in how to communicate a diagnosis and provision of information on available sources of information, such as in this video produced by patient organisations: https://www.youtube.com/watch?v=9DBA5D5Mx74 and Supplementary Material. In addition, tailoring information to patient needs to make it most appropriate is likely to improve patient satisfaction with the experience of the initial diagnostic consultation.

## Electronic supplementary material

Below is the link to the electronic supplementary material.

Supplementary material 1 (DOCX 34 KB)

Supplementary material 2 (PDF 188 KB)

Supplementary material 3 (MP4 4,14,026 KB)

Supplementary material 4 (MP4 23,019 KB)

## References

[1] Global Parkinson’s Disease Study Steering Committee (2002). Factors impacting on quality of life in Parkinson’s disease: results from an international survey. Mov Disord.

[2] Plouvier AOA, Olde Hartman TC, de Bont OA, Maandag S, Bloem BR, van WC, Lagro-Janssen ALM (2017). The diagnostic pathway of Parkinson’s disease: a cross-sectional survey study of factors influencing patient dissatisfaction. BMC Fam Pract.

[3] Stewart DA (2007). NICE guideline for Parkinson’s disease. Age Ageing.

[4] Ferreira JJ, Katzenschlager R, Bloem BR, Bonuccelli U, Burn D, Deuschl G, Dietrichs E, Fabbrini G, Friedman A, Kanovsky P, Kostic V, Nieuwboer A, Odin P, Poewe W, Rascol O, Sampaio C, Schupbach M, Tolosa E, Trenkwalder C, Schapira A, Berardelli A, Oertel WH (2013). Summary of the recommendations of the EFNS/MDS-ES review on therapeutic management of Parkinson’s disease. Eur J Neurol.

[5] Kim MK, Alvi A (1999). Breaking the bad news of cancer: the patient’s perspective. Laryngoscope.

[6] Fallowfield L, Jenkins V (2004). Communicating sad, bad, and difficult news in medicine. Lancet.

[7] Lemmon ME, Strowd RE (2016). Right brain: breaking bad news: communication education for neurology trainees. Neurology.

[8] Watling CJ, Brown JB (2007). Education research: communication skills for neurology residents: structured teaching and reflective practice. Neurology.

[9] McCluskey L, Casarett D, Siderowf A (2004). Breaking the news: a survey of ALS patients and their caregivers. Amyotroph Lateral Scler Other Motor Neuron Disord.

[10] Ford S (2007). Effectively communicating a diagnosis of Parkinson’s disease. Br J Hosp Med (Lond).

