# Supplementary material for: Patient experiences of receiving a diagnosis of Parkinson’s disease
Source: J Neurol. 2018 Mar 15;265(5):1151–7. doi: 10.1007/s00415-018-8817-8 (PMC5937885; doi:10.1007/s00415-018-8817-8)
Supplement: Supplementary file 1 — Supplementary material 1 (DOCX 34 KB) [file 415_2018_8817_MOESM1_ESM.docx]

**Supplementary material**

*Fig 1*. Explanation at first consultation (% of respondents, age of onset 50 and below)

Explanation at first consultation (% of respondents, patients who were diagnosed by a specialist PD neurologist only)

Explanation at first consultation (% of respondents, patients who were diagnosed by a general neurologist only)

Fig 2. Explanation at first consultation by country (% of respondents)

*Fig 3*. Healthcare professional who gave the diagnosis of PD by country (% of respondents)

*Fig 4*. Time given to ask questions at diagnosis by country (% of respondents)

Table 1. Patient satisfaction with the delivery of the initial diagnosis by country

|  | % Very satisfied/satisfied | % Neutral | % Very dissatisfied/dissatisfied |
| --- | --- | --- | --- |
| Hungary | 66 | 16 | 18 |
| Slovenia | 65 | 17 | 18 |
| Denmark | 63 | 20 | 17 |
| Holland | 57 | 28 | 15 |
| Sweden | 50 | 35 | 15 |
| UK | 46 | 25 | 29 |
| France | 40 | 34 | 26 |
| Germany | 37 | 38 | 25 |
| Spain | 34 | 32 | 34 |
| Italy | 19 | 9 | 72 |
