# Supplementary material for: Patient experiences of receiving a diagnosis of Parkinson’s disease
Source: J Neurol. 2018 Mar 15;265(5):1151–7. doi: 10.1007/s00415-018-8817-8 (PMC5937885; doi:10.1007/s00415-018-8817-8)
Supplement: Supplementary file 2 — Supplementary material 2 (PDF 188 KB) [file 415_2018_8817_MOESM2_ESM.pdf]

1. Introduction ©®™

This survey is part of My PD Journey, a multi-stakeholder project by the European Parkinson's Disease Association (EPDA).

With over 1.2 million people in Europe currently living with Parkinson's and this number forecast to double by 2030, My PD Journey aims to ensure that all care providers are coordinated and work together to remove the hurdles that prevent people with Parkinson's from receiving early and appropriate treatment and individualised care.

We would be most grateful if you would share your experiences of receiving a diagnosis, treatment and care for Parkinson's. This will help us understand how well healthcare systems are currently meeting the needs of people with Parkinson's and their families across Europe.

This survey should be completed by a person with Parkinson's disease, or by a family member / carer / healthcare professional on behalf of the person with Parkinson's. Any information you provide will be completely anonymous and treated in confidence.

The survey should take no more than 20 minutes. It is very important each person with Parkinson's completes the survey only once. This survey closes on 31 December 2014. If you have any questions about the survey, please email us at merrittrk@googlemail.com.

Thank you very much in advance for your time and help in completing this survey.

2. About you

1. In what year were you diagnosed with Parkinson's disease?

2. Are you male or female?

- ◯ Male
- ◯ Female

3. How would you describe the area that you live in?

- ◯ Rural
- ◯ Town
- ◯ City

4. Which country do you currently live in?

- ◯ Denmark
- ◯ France
- ◯ Germany
- ◯ Hungary
- ◯ Ireland
- ◯ Italy
- ◯ Netherlands
- ◯ Slovenia
- ◯ Spain
- ◯ Sweden
- ◯ United Kingdom

5. Are you currently employed?

- ◯ Yes
- ◯ No

6. What is/was your occupation?

13. Which of the following statements best describes how you feel about your independence?

- ◯ I am able to do all chores without slowness, difficulty or impairment
- ◯ I am able to do all chores with some degree of slowness, difficulty and impairment, and am beginning to be aware of difficulty
- ◯ Chores take twice as long and I am conscious of difficulty and slowness
- ◯ Chores take three to four times as long and I spend a large part of the day doing these
- ◯ I can do most chores, but exceedingly slowly and requiring a lot of effort
- ◯ I need help with half the chores and have difficulty with everything
- ◯ I can assist with all the chores, but am only able to do a few on my own
- ◯ I can manage a few chores with some effort, but need a lot of help
- ◯ I do nothing on my own, but can be a slight help with some chores
- ◯ I am totally dependent and helpless
- ◯ I am bedridden

5. Getting a diagnosis of Parkinson's disease

14. How old were you when you were first diagnosed with Parkinson's?

Years ____________________

15. How long has it been since you were first diagnosed with Parkinson's?

- ◯ Less than 1 year
- ◯ At least 1 year but less than 2 years
- ◯ At least 2 years but less than 3 years
- ◯ At least 3 years but less than 5 years
- ◯ At least 5 years but less than 10 years
- ◯ More than 10 years

16. Which country were you living in when you were first diagnosed with Parkinson's?

- ◯ Denmark
- ◯ France
- ◯ Germany
- ◯ Hungary
- ◯ Ireland
- ◯ Italy
- ◯ Netherlands
- ◯ Slovenia
- ◯ Spain
- ◯ Sweden
- ◯ United Kingdom
- ◯ Other (please specify)

____

6. Getting a diagnosis of Parkinson's disease

18. How long was it before you sought medical help after you first noticed your symptoms?

- ◯ Less than 1 month
- ◯ At least 1 month but less than 3 months
- ◯ At least 3 months but less than 6 months
- ◯ At least 6 months but less than 12 months
- ◯ 12 months or more
- ◯ Cannot remember

19. What happened during your first appointment with a healthcare professional? (Please select ALL that apply)

- ☐ Discussed your general medical history
- ☐ Carried out a physical examination
- ☐ Observed your symptom(s)
- ☐ Referred you to a specialist, or another doctor / healthcare professional
- ☐ Said nothing was wrong
- ☐ Said it was too early to tell if anything was wrong
- ☐ Said something was wrong, but not sure what
- ☐ Prescribed medication to relieve your symptom(s)
- ☐ Explained that you may have Parkinson's
- ☐ Explained that you may have another disease / condition
- ☐ Other (please specify)

8. Getting a diagnosis of Parkinson's disease

20. If you were referred to another healthcare professional, how long did it take before you saw them? (Please select ONE option in EACH row)

	Within 1 month	At least 1 month but less than 2 months	At least 2 months but less than 3 months	At least 3 months but less than 4 months	4 months or more	Cannot remember	Does not apply
General neurologist	◯	◯	◯	◯	◯	◯	◯
Neurologist who is a specialist in Parkinson's	◯	◯	◯	◯	◯	◯	◯
Care of the elderly doctor (geriatrician)	◯	◯	◯	◯	◯	◯	◯
Parkinson's disease nurse specialist	◯	◯	◯	◯	◯	◯	◯
Physiotherapist	◯	◯	◯	◯	◯	◯	◯
Occupational therapist	◯	◯	◯	◯	◯	◯	◯
Speech and language therapist	◯	◯	◯	◯	◯	◯	◯

21. What examinations or tests were carried out to confirm if you had Parkinson's? (Please select ALL that apply)

- ☐ General medical history
- ☐ Physical examination
- ☐ Observation of your symptoms
- ☐ Blood or urine tests
- ☐ Brain scan (e.g. CT, MRI, DaTSCAN)
- ☐ Genetic testing
- ☐ Other (please specify)

9. Getting a diagnosis of Parkinson's disease

22. Who gave you the diagnosis of Parkinson's?

- ◯ General practitioner or family doctor
- ◯ Hospital doctor
- ◯ General neurologist
- ◯ Neurologist who is a specialist in Parkinson's
- ◯ Care of the elderly doctor (geriatrician)
- ◯ Cannot remember
- ◯ Other (please specify)

23. How sensitively were you told you had Parkinson's?

Very sensitively	Quite sensitively	Not very sensitively	Not at all sensitively	Cannot remember
○	○	○	○	○

24. How satisfied were you with the consultation when the initial diagnosis was given?

Very satisfied	Satisfied	Neutral	Dissatisfied	Very dissatisfied	Cannot remember
○	○	○	○	○	○

10. Getting a diagnosis of Parkinson's disease

25. When you were given your diagnosis, what information were you given? (Please select ONE option for EACH row)

	Leaflet/handouts/signposting to online information	Explained verbally	Both handout and verbal information	I did not want any information	No information was provided	Cannot remember
Symptoms, diagnosis and causes of Parkinson's	○	○	○	○	○	○
Medication	○	○	○	○	○	○
Surgical treatments	○	○	○	○	○	○
Non-drug treatments (e.g. physiotherapy, occupational therapy, complementary therapies etc.)	○	○	○	○	○	○
Maintaining physical well being (e.g. healthy eating, exercise etc.)	○	○	○	○	○	○
Maintaining emotional well being (e.g. anxiety, mood changes etc.)	○	○	○	○	○	○
Financial help available	○	○	○	○	○	○
Support organisations (e.g. patient associations)	○	○	○	○	○	○
Support for carers	○	○	○	○	○	○
Where to find more information on Parkinson's	○	○	○	○	○	○
Taking part in clinical trials	○	○	○	○	○	○

26. If you were given information, how helpful was it?

Very helpful	Quite helpful	Not very helpful	Not helpful	Cannot remember	No information was provided
○	○	○	○	○	○

11. Getting a diagnosis of Parkinson's disease

27. When you were given your diagnosis of Parkinson's, were you given time to ask questions and discuss your concerns?

- ◯ Yes, I was given enough time
- ◯ Yes, but I would have liked more time
- ◯ No, I was not given any time
- ◯ I did not want to ask questions at that time
- ◯ I did not feel able to ask questions or discuss concerns at that time
- ◯ Cannot remember

12. Your treatment

28. How soon after your diagnosis did you receive any medication or treatment therapy?

- ◯ This was discussed but I decided against taking medication at that time
- ◯ Immediately
- ◯ Less than 1 month
- ◯ At least 1 month but less than 3 months
- ◯ At least 3 months but less than 6 months
- ◯ At least 6 months but less than 12 months
- ◯ 12 months or more
- ◯ Cannot remember
